# Executive compensation controls and corporate cash holdings

**DOI:** 10.1371/journal.pone.0285387

**Published:** 2023-09-08

**Authors:** Peiqiong Wang, Xianhua Zhang, Taozhi Wang, Zilu Wan

**Affiliations:** School of Accounting, Lanzhou University of Finance and Economics, Lanzhou City, Gansu Province, China; Bucharest University of Economic Studies: Academia de Studii Economice din Bucuresti, ROMANIA

## Abstract

As a crucial component of internal corporate governance, remuneration controls possess the potential to influence the cash holdings of firms. However, identifying the causal relationship between these controls and such holdings presents a considerable challenge. To address this research gap, this paper leverages the implementation of China’s Guidance on Further Regulating the Remuneration Management of Heads of Central Enterprises as a quasi-natural experiment to investigate the relationship between executive remuneration controls and firms’ cash holdings, utilizing a double-difference approach. Based on an analysis of a sample of listed companies from 2007–2012, the results indicate that firms subject to regulated executive compensation exhibit lower cash holdings. To ensure the robustness of these findings, various statistical techniques such as parallel trend tests, variable replacement, propensity score matching, and placebo tests were employed. Additionally, a mechanism test was conducted, whereby the mediating effect of executive compensation controls on firms’ cash holdings was examined, revealing a reduction in internal agency costs. Finally, the analysis of heterogeneity demonstrated that the impact of executive compensation controls on firms’ cash holdings was more pronounced in companies with high-quality internal controls, stronger management oversight, and lower information asymmetry.

## 1. Introduction

The phenomenon of income disparity within a company has been a critical area of research in the domain of corporate governance [1, 2]. Previous research has demonstrated that the pay disparity within a firm can result in a motivational impact on employees, with two prominent theories that aim to explain the underlying mechanisms: the tournament theory and the social comparison theory [1, 3–7]. The tournament theory posits that the difference in pay between employees can spur inter-employee competition and comparison, with a larger disparity in pay between adjacent levels leading to a greater motivational impact and a subsequent improvement in the firm’s operational performance [1]. This theory has received support from numerous empirical studies [8–10]. Furthermore, Mehran noted that executive pay serves as one of the key drivers for enhancing business performance [11]. However, there are also views that excessive pay may hurt employee job motivation. Some studies, rooted in the social comparison theory, have investigated the issue of pay disparity, such as the fairness theory, which states that an individual’s emotions and behavior are influenced by the extent of fairness in their income relative to others, regardless of whether the comparison is other-regarding or self-regarding [12, 13]. The theory of organizational politics posits that excessive pay disparities within an organization can have negative consequences for both pay motivation and overall productivity. Specifically, it suggests that employees with lower pay levels may experience a sense of exploitation, which can lead to decreased cooperation with others and an increase in self-serving behaviors [4]. This in turn can seriously undermine the effectiveness of pay as a motivator. Furthermore, research by Hayward and Hambrick found that when the pay of managers is significantly higher than that of other employees, it can result in overconfidence among managers and contribute to suboptimal decision-making [14]. To mitigate these effects, many organizations implement pay control measures to constrain excessive pay disparities.

The regulation of salaries has been shown to effectively align the operational goals of managers with those of the company owners, balancing personal and corporate interests [15]. This assertion has been supported by academic research which has demonstrated a limited correlation between executive salaries and corporate performance [16]. Although salary incentives can be beneficial, it’s important to bear in mind that certain industries may have limitations on their effectiveness due to inherent characteristics. Additionally, excessive regulation of salaries can result in negative consequences for a company’s management. For instance, Thanassoulis found through his research on salary regulation in the banking industry that such regulation can be detrimental and drive up managerial salaries through internal competition [17]. Similarly, Frydman and Molloy evaluated the efficacy of the Salary Limitation Order policy imposed by the Roosevelt administration during World War II, concluding that moderate restrictions are preferable, as overly stringent regulation may undermine the policy’s success [18]. Overall, the academic community acknowledges the impact of salary regulation on corporate governance and, from an incomplete contract theory perspective [19], posits that executives may exhibit self-serving behavior in response to higher salaries, which can be effectively countered through the implementation of salary regulation, thereby reducing agency costs.

Governments across the globe have made efforts to control the salaries of top executives. For instance, the Obama administration in the United States took a strong stance on executive compensation and imposed a Salary Limitation Order of $500,000 on senior management of companies receiving government "special assistance". The Federal Reserve mandated that thousands of banking institutions, including bank holding companies and state banks, submit their compensation plans for approval [20]. The Chinese government recognizes the negative effects that excessive salary disparities can have on businesses. As a result, in September 2009, The Chinese government has recently released the "Guidance on Further Regulating the Salaries of Central Enterprise Officials" to standardize the salary distribution of senior managers in state-owned enterprises. The "Guidance" sets a maximum salary limit of 12 times the average salary of employees, with an upper limit of 600,000 yuan per year for senior executives in central enterprises. To ensure reasonable and appropriate salary structures, the "Guidance" emphasizes the importance of combining market regulation and government oversight. It also provides clear regulations on the salary management of senior managers in central state-owned enterprises, including the scope of application, fundamental principles, salary structure, and level. This initiative aims to promote standardized and regulated management practices in state-owned enterprises.

Regulating executive compensation can have significant impacts on a firm’s cash holdings, as shown by the monetary demand theory. Firms hold cash for various reasons, including transactional, speculative, and precautionary motives. Precautionary motives are particularly relevant as they often drive firms to maintain significant cash reserves [21–23]. According to Bates et al., American companies’ cash holdings continued to increase in the late 20th century, primarily due to precautionary motives [22]. However, cash held for precautionary purposes is one of the most liquid and readily disposable assets within the firm and can be converted into personal income for executives at relatively low costs compared to other liquid assets [24]. This presents a particular challenge in the case of state-owned enterprises in China, where high agency costs and a lack of internal control result in asymmetric information and increased opportunities for rent-seeking behavior by executives [24, 25]. These agency costs can facilitate self-interested behavior by management, leading to excessive compensation and in-service consumption, which can influence the level of cash holdings within the firm. As such, regulating executive compensation is crucial to ensuring that the compensation structures of senior managers in state-owned enterprises are reasonable, levels are appropriate, and management is standardized, ultimately promoting the efficient use of corporate resources. Based on management theory, it can be argued that providing excessive compensation to management is tantamount to endowing them with a greater amount of power. Additionally, the presence of cash can serve as a major incentive for rent-seeking behavior within a company. Thus, implementing compensation regulation measures represents a viable approach to curbing management power and limiting the amount of cash held within the organization. Such measures have the potential to not only reduce holding costs but also to lower agency costs, as outlined by Ni X in a recent publication [26].

The impact of salary regulation on a company’s cash holdings has not been extensively studied in the existing literature. This paper posits that governance mechanisms have consistently served as a crucial avenue for reducing agency costs within organizations, with the regulation of executive salaries being a salient aspect of contemporary corporate governance. By limiting salary levels to reasonable levels, the agency costs between owners and managers can be reduced, while not impeding the motivation of top executives. On the other hand, restrictions on excessive salaries can also serve as a deterrent for managers, helping to mitigate rent-seeking behavior driven by personal interests. In China, the regulation of salaries for executives in state-owned enterprises has been effective in curtailing excessive salaries, suppressing self-interested behavior, and reducing agency costs and excessive cash holding levels within these enterprises. Additionally, the implementation of salary control policies can act as a deterrent to power-seeking behavior by executives, compelling managers to improve their operational proficiency. Excessive corporate free cash flow can also hinder operational efficiency, and as such, managers have the incentive to optimize their cash holding level and reduce corporate free cash flow to further enhance operational efficiency.

This article explores the impact of salary regulation on cash holding levels in Chinese listed companies, utilizing the "Guiding Opinions" issued by the Chinese government in September 2009 as a natural experiment. By employing a double difference model, the study reveals that the implementation of salary regulation significantly reduced cash holding levels in state-owned enterprises in China. This finding holds after conducting robustness checks, and the study further shows that the suppression effect of salary regulation on cash holding levels is more prominent in firms with weaker internal controls, greater management supervision, and higher levels of information opacity. Moreover, the study indicates that salary regulation can reduce agency costs and subsequently decrease a company’s cash holding level.

The following points make up the majority of this paper’s contributions.

Firstly, this study utilizes the "Guiding Opinions" as a basis to investigate the distinctive governance mechanism of Chinese state-owned enterprises and its economic implications through a quasi-natural experiment. In contrast to existing research, which predominantly focuses on cash holdings from the perspective of liquidity demand, this article analyzes the impact of compensation control on corporate cash holdings and its underlying mechanism from the perspectives of corporate governance and executive self-interest. As such, this study contributes to the literature on compensation control and cash holdings by providing a novel analytical framework. Secondly, to assess the policy’s impact, this study employs a difference-in-differences model. In contrast to other models, the difference-in-differences model effectively addresses the endogeneity of this study’s research questions, thereby offering methodological innovation. Thirdly, concerning policy research, this study specifically selects the compensation control policy implemented in 2009, which distinguishes itself from other policies such as the 18th National Congress in 2012 and the Second Salary Limitation Order in 2015. The selection of this policy is less susceptible to the influence of other policy factors, thus facilitating a more accurate evaluation of its effects and providing a superior research context for the academic community. Finally, this study examines how compensation control reduces corporate cash holdings from the perspective of policy impact by influencing management self-interest and reducing internal agency costs. This line of analysis enriches the research scope of compensation control, providing a basis for future scholars to expand on this topic.

The upcoming sections of this essay are structured as follows: Part II focuses on theoretical research and hypothesis development, while Part III describes the data and variable hypotheses. Part IV presents the empirical study analysis, and Part V conducts the mechanism test and heterogeneity test. Finally, Part VI concludes the entire study.

## 2. Theoretical study and hypothesis formulation

### 2.1. Compensation governance and corporate cash holdings

Because of how modern corporate systems are set up, operation and ownership must be separated, which creates significant agency issues and expenses for the company [27]. Excessive incentive compensation packages increase agency costs and encourage CEOs to act in their self-interest. For their benefit, executives have incentives to raise the company’s cash holdings, which include the following. First, managers can turn capital kept within the company into their gains by using lower costs [24]. According to Jensen, a significant portion of an executive’s on-the-job expenses come from the company’s cash [28]. When companies create executive compensation packages, they typically do not factor in the firm’s cash holdings at the time of the compensation formulation program. However, they do factor them in at the time of the compensation assessment system when deciding on compensation contracts later on based on the firm’s operating conditions [29]. Therefore, to maximize their payoffs, executives will be self-interestedly motivated to boost the firm’s cash holding level after the accounting period. Due to the fact that rational managers prioritize maximizing their own utility over maximizing shareholder value, in contrast to principal shareholders, and due to the expensive cost of regulation, cash is more likely to be appropriated by the company’s management [28]. Second, having a lot of cash also makes it easier for corporate executives to spend money on the job, which drives up the firm’s agency fees. Agency costs increase the likelihood that managers will utilize excessive investment, financial mismanagement, company empire-building, and on-the-job spending to extort shareholders’ equity when firms have higher free cash flow [22, 25, 30]. Higher information asymmetry [31], problems in corporate governance [32, 33], and worse investor protection are all associated with this phenomenon in particular organizations [34]. Particularly in companies with lesser levels of investor protection, this is apparent [34]. There exist studies indicating that inadequate or excessive incentives for cash holdings among corporations can lead to the utilization of company funds by executives for frivolous expenditures and inefficient investments, thereby diminishing the company’s cash reserves [35]. Nonetheless, a high level of cash holdings does not necessarily entail unfavorable outcomes. Recent research has demonstrated that the accumulation of a greater quantity of cash prior to a financial crisis can aid small and medium-sized foreign enterprises in sustaining or elevating their profitability during periods of economic turmoil [36].

The principal-agent perspective suggests that agency conflicts may cause the level of cash holdings to vary between being high under the flexibility hypothesis and low under the dissipation hypothesis [33]. We contend that the flexible hypothesis allows management to retain more cash assets for self-interested uses like on-the-job spending and benefits appropriation. We also contend that compensation controls can have a governance effect by limiting management’s agency incentives to appropriate cash, thereby lowering the level of corporate cash holdings. Nonetheless, extant literature suggests that when management holds equity, the decision-making process may be influenced by their aspiration to maximize personal benefits, which may come at the expense of the company and its shareholders’ interests. This could lead to an increase in agency costs and a decrease in the company’s attempts to secure external financing while augmenting its cash reserves [35]. Essentially, robust corporate governance mechanisms can effectively curb the adverse effects of agency conflicts, leading to a decrease in the level of cash holdings of the company. However, it is worth noting that if certain equity-holding executives prioritize their own interests, it may lead to an escalation of agency costs and the level of cash holdings of the company. In other words, an efficient corporate governance process will effectively minimize the amount of cash kept by the company by reducing the negative impacts of agency conflicts. Contrarily, the limitation of compensation control also sends warning signals to SOE executives, motivating them to focus more effort on improving how well the company is run. By cutting agency costs and utilizing the additional funds for company development, firms can reduce their level of cash reserves, thereby highlighting the strong link between agency conflicts and the value of cash holdings. On the other side, Faulkender and Wang examined the issue from the standpoint of financial features and discovered that growing cash holdings causes a decline in the firm’s marginal value [37]. In addition to the two aforementioned hypotheses, other studies have shown that a company’s cash holdings can encourage executives to extract rent in the form of higher executive compensation. Executives can use their cash holdings as bargaining chips in negotiations with the company after the fact. Therefore, cash holdings create a form of direct agency cost that goes beyond the areas of the corporate building and investment [29].

### 2.2. Hypothesis formulation

This article examines how compensation regulations can reduce a company’s cash holdings by impacting management’s self-interest and internal agency costs, through the lens of policy shocks.

The Chinese government’s implementation of a "Salary Limitation Order" for state-owned enterprises has several potential effects. Firstly, it can reasonably limit excessive and unreasonable executive compensation, aligning it with operational performance. Secondly, the cap can mitigate adverse effects stemming from resource mismatches, avoiding executives’ short-sighted behavior that targets short-term compensation goals, aligning management and owners’ interests, reducing agency costs, and further lowering cash holdings. Thirdly, state-owned enterprise executives’ greater management power may lead to unequal compensation contracts and other self-interested behavior. The Chinese government’s "Guiding Opinions" restricts the further expansion of management power, effectively curbing self-interested behavior, and further reducing cash holdings. Fourthly, compensation regulation incentivizes high-level executives to improve operational performance rather than seek self-satisfaction, thereby greatly curbing self-interested behavior among state-owned enterprise executives and reducing cash holdings.

Based on our analysis, this study posits that the "Salary Limitation Order" can mitigate self-interested behavior among state-owned enterprise executives. Cash, as one of the most liquid and easily managed assets, frequently reflects such behavior. The more severe the agency problem within a company, the greater the risk of misappropriation by management [38]. Due to the political incentives that state-owned enterprise executives often receive, lower sensitivity to performance-based pay may be effective because political incentives can replace monetary incentives [39, 40]. Research has indicated that the impact of pay-performance sensitivity on a company’s level of cash holdings is equally important for state-owned enterprises and non-state-owned enterprises. Moreover, the correlation between pay-performance sensitivity and company performance is even stronger for state-owned enterprises than for non-state-owned enterprises. In fact, the effect of executive pay incentives on improving company performance is at least as significant as that of non-state-owned enterprises [41]. The Chinese government’s "Guiding Opinions" introduce salary control measures that limit incentives and align management’s interests with those of shareholders. This helps to reduce agency costs in the enterprise. Furthermore, it regulates the expansion of managerial authority through government intervention, thereby suppressing self-interested behavior in state-owned enterprises.

In conclusion, these two factors demonstrate that salary control can shrink the scope of self-interested behavior among state-owned enterprise managers by decreasing agency costs. Optimizing cash holdings and reducing state-owned enterprises’ cash reserves will improve resource allocation efficiency. This analysis leads to two proposed hypotheses: H1a and Competitive assumptions H1b

H1a: The implementation of the "Salary Limitation Order" policy will significantly decrease cash holdings in state-owned enterprises.
H1b: The implementation of the "Salary Limitation Order" policy will significantly increase cash holdings in state-owned enterprises.

Higher agency costs result in increased self-interest space for a company’s management. As a consequence, the level of cash holdings will also be higher. Therefore, agency costs are an important factor that affects a company’s level of cash holdings by influencing self-interest motivations. The management expense ratio is defined as the ratio of a company’s management expenses to its main business income and is commonly used as a measure of agency costs. The higher the management expense ratio, the greater the likelihood of self-interested behavior by the company’s management. To test whether agency costs play an intermediary role, this paper conducts a mechanism test using the management expense ratio as one of the indicators of agency costs and proposes the following hypothesis:

H2: The implementation of the "Salary Limitation Order" results in agency costs playing an intermediary role in reducing a company’s level of cash holdings.

## 3. Data description and variable definitions

### 3.1. Data sources

This article examines a sample of listed companies in China. First, this paper chooses 2007–2012 as the research interval to conform to the standard difference-difference model, which is used to evaluate the changes in the first three years and the last three years of this policy. Second, the research interval is due to the end of 2012, because at the end of 2012, China held the 18th National Congress of the Communist Party of China, which adopted many policies and documents related to corruption governance and state-owned enterprise management, which would have a great impact on the research conclusions of this paper. Therefore, the research interval of this paper is due to 2012. The data utilized in this study primarily originates from the CSMAR database in China. During the data processing phase, several screening procedures were implemented to ensure the validity of the data: (1) Given the unique characteristics of the financial and insurance industries in China, companies in these sectors were excluded; (2) Any samples that received delisting warnings were removed, as they may have experienced financial crises and impacted the accuracy of the research results; (3) Samples with missing values for the key regression variables were eliminated. Following these procedures, a total of 5926 valid company-year observations were obtained. To reduce the impact of extreme values on the empirical results, all continuous variables were trimmed at the 1st and 99th percentiles.

### 3.2. Definition of variables

#### 3.2.1. Salary control (*Treat*_*i*_ * *Post*_*t*_)

Given the exogenous nature of the "Guiding Opinions" policy to the operational decision-making of enterprises, and its differentiation of all listed companies in China into a salary control group (consisting of all state-owned enterprises) and a non-salary control group (comprising non-state-owned enterprises), it presents a valuable research scenario for us to assess the policy’s effects. To mitigate potential endogeneity concerns, this study employs the standard difference-in-differences (DID) model variable setting and defines the policy dummy variable Treat_i_ as the set of state-owned enterprises (SOE) subjected to salary control. The policy’s implementation date is established as 2009, and we define the time dummy variable Post_t_ to be 0 prior to 2009 and 1 for the years after and including 2009.

#### 3.2.2. Cash holding (*Cash*_*i*,*t*_)

With regard to the measurement of this indicator, this study draws on the methodology employed by Ozkan and Ozkan by utilizing the formula of "Cash and Cash Equivalents/Total Assets" [42]. A higher value of this indicator indicates a larger amount of cash held by the enterprise. To mitigate the influence of cash on total assets, the study further references the approaches of Harford et al., Kalcheva and Lins, etc. [33, 34], and adopts the form of "(Cash + Liquid Financial Assets)/(Total Assets—Cash)" as a supplementary (*Cash_a*_*i*,*t*_) to the interpretive variable in the study, thereby enhancing the robustness of its conclusions.

#### 3.2.3. Control variables (*Controls*_*i*,*t*_)

With the aim of mitigating the impact of other potential factors on a corporation’s cash holdings, this study references the methodologies employed by Nyborg and Wang, Begenau and Palazzo, and Eskandari and Zamanian [43–45] and selects the following variables as control variables: company size (Size), financial leverage (Lev), profitability (Roa), company growth (Growth), audit quality (Audit), board size (Board), executive compensation (Wage3), and equity concentration (Top1). Table 1 contains definitions of the main variables used in this study.

**Table 1:** Variable description.

Variable	Description
*Cash*_*i*,*t*_	Corporate cash holding levels, Cash and cash equivalents/total assets
*Cash_a*_*i*,*t*_	Corporate cash holding levels, (Monetary funds + financial assets held for trading)/(Total assets—cash)
*TP*_*i*,*t*_	Compensation control, interactive term, calculated as *SOE*_*i*,*t*_ * *Post*_*t*_
*SOE*_*i*,*t*_	Reflects whether it is a state-owned enterprise, state-owned enterprise *SOE*_*i*,*t*_ takes 1, non-state-owned enterprise *SOE*_*i*,*t*_ takes 0
*Post*_*t*_	2009 and thereafter *Post*_*t*_ = 1, and conversely, *Post*_*t*_ = 0
*Size*_*i*,*t*_	Company size was calculated as the natural logarithm of total assets at the year-end
*Lev*_*i*,*t*_	Financial leverage, measured by current liability ratio
*ROA*_*i*,*t*_	Profitability, measured by return on total assets
*Growth*_*i*,*t*_	Company growth, measured as (current period operating revenue—previous period operating revenue)/previous period operating revenue
*Audit*_*i*,*t*_	Audit quality, *Audit*_*i*,*t*_ takes the value of 1 when a standard unqualified audit opinion is obtained, otherwise it is 0
*Board*_*i*,*t*_	Board size, measured as ln(1 + number of board members)
*Wage*3_*i*,*t*_	Executive compensation, measured as the natural logarithm of the sum of the top three executives’ compensation
*TOP*1_*i*,*t*_	The concentration of shareholding, measured by the percentage of shares held by the largest shareholder

### 3.3. Research model

To assess the influence of regulatory oversight of executive compensation on corporate cash holdings, this study leverages the implementation of the "Guiding Opinions" as a natural experiment, drawing on the methodology of Opler et al. to formulate the following model (1) [21]:

$$Cash_{i,t}/Cash\_a_{i,t}=\beta_{0}+\beta_{1}Treat_{i}\times Post_{t}+\beta_{2}Treat_{i}+\beta_{i}Controls_{i,t}+\sum Year+\sum Industry+\varepsilon_{i,t}$$ (1)

The data used in this paper is a panel data model, and due to the availability of data, the data used is unbalanced panel data. In Model (1), variable *Cash*_*i*,*t*_/*Cash*_*a*_*i*,*t*_ signifies the cash holdings level of firm i in year t. The primary explanatory variable, represented by the interaction between group and time dummy variables, is denoted by *Treat*_*i*_ × *Post*_*t*_ and is the primary focus of this study. The group dummy variable, represented by *Treat*_*i*_, categorizes state-owned enterprises subjected to compensation regulation as the experimental group, while non-state-owned enterprises serve as the control group. The time dummy variable, represented by *Post*_*t*_, is based on the implementation of the "Guidance," 2009 and thereafter *Post*_*t*_ are defined as 1 and the years before 2009 are defined as 0. Variable *Controls*_*i*,*t*_ represents a control variable, encompassing additional factors affecting cash holdings, while ∑*Year* represents annual fixed effects, ∑*Industry* represents industry fixed effects, and *ε*_*i*,*t*_ represents the error term. Due to the control of annual fixed effects, Post_t_ has been omitted from the model.

The principal objective of this paper is to examine the regression coefficient *β*1 of *TP*_*i*,*t*_(*Treat*_*i*_ × *Post*_*t*_), which sheds light on the overall effect of compensation control on corporate cash holdings. If the coefficient *β*1 in the regression outcome is significantly negative, it attests to the support of hypothesis H1, indicating that, relative to enterprises not subject to compensation control, compensation control exerts a significantly detrimental impact on the cash holdings of controlled enterprises.

## 4. Empirical analysis study

### 4.1. Descriptive statistics

Table 2 displays the descriptive statistics of the central variables in the present study, including the mean, median, standard deviation, minimum, and maximum values. The results reveal that, for the sample analyzed, the measures of cash holdings (*Cash*_*i*,*t*_/*Cash*_*a*_*i*,*t*_) have minimum values of 0.0146 and 0.0125, and maximum values of 0.6886 and 2.1238, respectively. These figures underscore the considerable variation in the amount of cash held by listed firms in China. The median, at 0.1558 and 0.1594, suggest that the sample’s cash holdings proportion is 15.58% and 15.94%, respectively, indicating that the analyzed companies hold a relatively high level of cash holdings.

**Table 2:** Descriptive statistics.

Variable	N	Mean	p50	SD	Min	Max
*Cash*_*i*,*t*_	5926	0.1900	0.1558	0.1323	0.0146	0.6886
*Cash_a*_*i*,*t*_	5926	0.2552	0.1594	0.2937	0.0125	2.1238
*TP*_*i*,*t*_	5926	0.3606	0	0.4802	0	1
*SOE*_*i*,*t*_	5926	0.5216	1	0.4996	0	1
*Size*_*i*,*t*_	5926	21.9027	21.7266	1.2117	19.6829	25.8100
*Lev*_*i*,*t*_	5926	0.8332	0.8985	0.1792	0.2615	1
*ROA*_*i*,*t*_	5926	0.0437	0.0392	0.0512	-0.1407	0.2078
*Growth*_*i*,*t*_	5926	0.0021	0.0014	0.0044	-0.0053	0.0282
*Audit*_*i*,*t*_	5926	0.9804	1	0.1385	0	1
*Board*_*i*,*t*_	5926	2.1841	2.1972	0.1944	1.6094	2.7081
*Wage*3_*i*,*t*_	5926	13.9404	13.9581	0.7377	12.1007	15.7718
*TOP*1_*i*,*t*_	5926	36.9268	35.5400	15.2526	8.9300	74.9600

### 4.2. Correlation analysis

According to the summary in Table 3, which presents the results of the correlation analysis conducted on the key variables in this study, we can conclude that there is a negative correlation among firm *Size*_*i*,*t*_, *Board*_*i*,*t*_, and *Cash*_*i*,*t*_/*Cash*_*a*_*i*,*t*_. In other words, as the size of the firm and the board increases, the amount of cash held by the firm decreases. *ROA*_*i*,*t*_, *Audit*_*i*,*t*_, *Wage*3_*i*,*t*_, *TOP*1_*i*,*t*_, and *Cash*_*i*,*t*_/*Cash*_*a*_*i*,*t*_ all have positive relationships, meaning that the more profitable a company is and the higher the audit quality, the more cash it needs and the higher the amount of cash it has on hand, which is more in line with previous studies [43, 46, 47]. The low correlation coefficient values between each explanatory variable and the other variables, however, show that multicollinearity between the variables is not a significant issue.

**Table 3:** Correlation analysis.

	*Cash*_*i*,*t*_	*Cash_a*_*i*,*t*_	*TP*_*i*,*t*_	*SOE*_*i*,*t*_	*Size*_*i*,*t*_	*Lev*_*i*,*t*_	*ROA*_*i*,*t*_	*Growth*_*i*,*t*_	*Audit*_*i*,*t*_	*Board*_*i*,*t*_	*Wage*3_*i*,*t*_	*TOP*1_*i*,*t*_
*Cash*_*i*,*t*_	1											
*Cash_a*_*i*,*t*_	0.913***	1										
*TP*_*i*,*t*_	-0.091***	-0.080***	1									
*SOE*_*i*,*t*_	-0.183***	-0.151***	0.719***	1								
*Size*_*i*,*t*_	-0.166***	-0.165***	0.334***	0.325***	1							
*Lev*_*i*,*t*_	0.245***	0.186***	-0.188***	-0.169***	-0.361***	1						
*ROA*_*i*,*t*_	0.275***	0.260***	-0.060***	-0.101***	0.035***	0.076***	1					
*Growth*_*i*,*t*_	0.002	-0.003	-0.025*	-0.011	0.093***	-0.0170	0.222***	1				
*Audit*_*i*,*t*_	0.067***	0.054***	-0.003	-0.016	0.068***	-0.041***	0.183***	0.009	1			
*Board*_*i*,*t*_	-0.059***	-0.064***	0.150***	0.221***	0.275***	-0.120***	0.009	-0.005	0.008	1		
*Wage*3_*i*,*t*_	0.113***	0.096***	0.188***	0.088***	0.460***	-0.128***	0.286***	0.055***	0.093***	0.157***	1	
*TOP*1_*i*,*t*_	0.037***	0.039***	0.108***	0.124***	0.250***	-0.073***	0.088***	0.088***	0.048***	-0.041***	0.042***	1

Note:

***, **, and * indicate significance at the 1%, 5%, and 10% levels.

### 4.3. Baseline regression

This study utilized stepwise regression analysis to test hypothesis H, focusing on the coefficient of the interaction term *TP*_*i*,*t*_ in the regression results. The findings are summarized in Table 4. Columns (1) and (3) show the results when adjusting only for year and industry-fixed effects, respectively, without additional control variables. The coefficient of the interaction term *TP*_*i*,*t*_ is significant at the 1% level, with a value of -0.013. Columns (2) and (4) display the results of regression analyses without year and firm fixed effects and other control variables, with coefficients of the interaction term *TP*_*i*,*t*_ of -0.0347 and -0.0855, respectively, both substantially negative at the 1% level. When accounting for the year and industry-level effects and all control variables, the coefficients on the interaction term *TP*_*i*,*t*_ are substantially negative at the 1% level, with values of -0.0259 and -0.0661, in columns (2) and (4), respectively. These findings suggest that payroll restrictions significantly reduce state-owned enterprise (SOE) cash holdings compared to non-SOE cash holdings, supporting hypothesis H1a but not H1b. The findings of this study are in concurrence with the results reported by Dimitropoulos et al. and Cheng et al., thereby underscoring the consistency of our research outcomes with those of other studies conducted in different countries [29, 36]. This suggests that our findings are not exclusive to China but may hold true across a wider spectrum of geographic and cultural contexts. The "Salary Limitation Order" diminishes executives’ motivation to act in their self-interest, leading to lower agency costs of cash holdings and encouraging a reduction in cash holdings.

**Table 4:** Baseline regression.

	(1)	(2)	(3)	(4)
	*Cash*_*i*,*t*_	*Cash*_*i*,*t*_	*Cash_a*_*i*,*t*_	*Cash_a*_*i*,*t*_
*TP*_*i*,*t*_	-0.0347***	-0.0259***	-0.0855***	-0.0661***
	(-5.0989)	(-4.0523)	(-6.0054)	(-4.9160)
*SOE*_*i*,*t*_	-0.0134*	-0.0019	-0.0085	0.0199
	(-1.8915)	(-0.2741)	(-0.6520)	(1.5252)
*Size*_*i*,*t*_		-0.0191***		-0.0477***
		(-6.9411)		(-8.4951)
*Lev*_*i*,*t*_		0.1137***		0.1576***
		(7.8375)		(5.5036)
*ROA*_*i*,*t*_		0.5752***		1.2765***
		(10.1417)		(9.3622)
*Growth*_*i*,*t*_		-0.9837***		-2.6014***
		(-2.7012)		(-3.3822)
*Audit*_*i*,*t*_		0.0238*		0.0307
		(1.8493)		(1.4778)
*Board*_*i*,*t*_		0.0125		0.0088
		(0.9620)		(0.3223)
*Wage*3_*i*,*t*_		0.0201***		0.0397***
		(4.7467)		(4.4780)
*TOP*1_*i*,*t*_		0.0007***		0.0014***
		(3.4888)		(3.5745)
Cons	0.2095***	0.1464**	0.2905***	0.4777***
	(50.4557)	(2.0262)	(31.9026)	(3.3449)
N	5926	5926	5926	5926
Year	Yes	Yes	Yes	Yes
Industry	Yes	Yes	Yes	Yes
adj. R2	0.0843	0.1975	0.0586	0.1568

Note: The standard errors are corrected for clustering at the firm level.

***, **, and * indicate significance at the 1%, 5%, and 10% levels, respectively; t-values are given in parentheses.

### 4.4. Robustness test

The following robustness tests and discussions were carried out to further guarantee the accuracy of the paper’s conclusions.

#### 4.4.1. Test for parallel trends

To ensure the basic premise assumption of the double difference model, this study employs the event study method to perform a parallel trend analysis. Specifically, the experimental and control groups must exhibit a similar trend before the policy’s implementation. The study uses 2009 as the base year and generates year dummy variables and interaction terms (pre_2, pre_1, current, post_1, post_2 and post_3) between the policy variable (*SOE*_*i*,*t*_) and explanatory variables (*Cash*_*i*,*t*_/*Cash*_*a*_*i*,*t*_) to test the hypothesis. pre_2 and pre_1 refer to the two and one years before the policy implementation, while current, post_1, post_2, and post_3 represent the year of implementation and the subsequent years. The regression findings in Table 5 indicate that the coefficients of pre_2 and current are not significant, suggesting a similar trend of change between the experimental and control groups before the policy’s implementation. When the period before the policy is excluded as the base year, the parallel trend hypothesis is satisfied. The coefficients of post_1, post_2 and post_3 are all significantly negative at the 1% level, indicating that the "Salary Limitation Order" has a substantial moderating effect on the number of cash holdings.

**Table 5:** Parallel trend test.

	(1)	(2)
	*Cash*_*i*,*t*_	*Cash_a*_*i*,*t*_
pre_2	-0.0105	-0.0196
	(-1.5004)	(-1.5403)
current	0.0095	0.0056
	(1.4059)	(0.4097)
post_1	-0.0219***	-0.0566***
	(-2.6224)	(-2.9748)
post_2	-0.0456***	-0.1100***
	(-5.1069)	(-5.3745)
post_3	-0.0497***	-0.1095***
	(-5.8838)	(-6.1517)
*SOE*_*i*,*t*_	0.0029	0.0287**
	(0.3885)	(2.0171)
*Size*_*i*,*t*_	-0.0187***	-0.0468***
	(-6.7713)	(-8.3353)
*Lev*_*i*,*t*_	0.1136***	0.1572***
	(7.8473)	(5.5046)
*ROA*_*i*,*t*_	0.5859***	1.2977***
	(10.3490)	(9.5149)
*Growth*_*i*,*t*_	-1.0940***	-2.8200***
	(-3.0121)	(-3.6658)
*Audit*_*i*,*t*_	0.0221*	0.0272
	(1.7007)	(1.2857)
*Board*_*i*,*t*_	0.0122	0.0082
	(0.9441)	(0.3012)
*Wage*3_*i*,*t*_	0.0198***	0.0391***
	(4.6916)	(4.4288)
*TOP*1_*i*,*t*_	0.0006***	0.0014***
	(3.4531)	(3.5431)
Cons	0.1423**	0.4696***
	(1.9766)	(3.3038)
N	5926	5926
Year	Yes	Yes
Industry	Yes	Yes
adj. R2	0.2023	0.1607

Note: The standard errors are corrected for clustering at the firm level.

***, **, and * indicate significance at the 1%, 5%, and 10% levels, respectively; t-values are given in parentheses.

**Fig 1:**
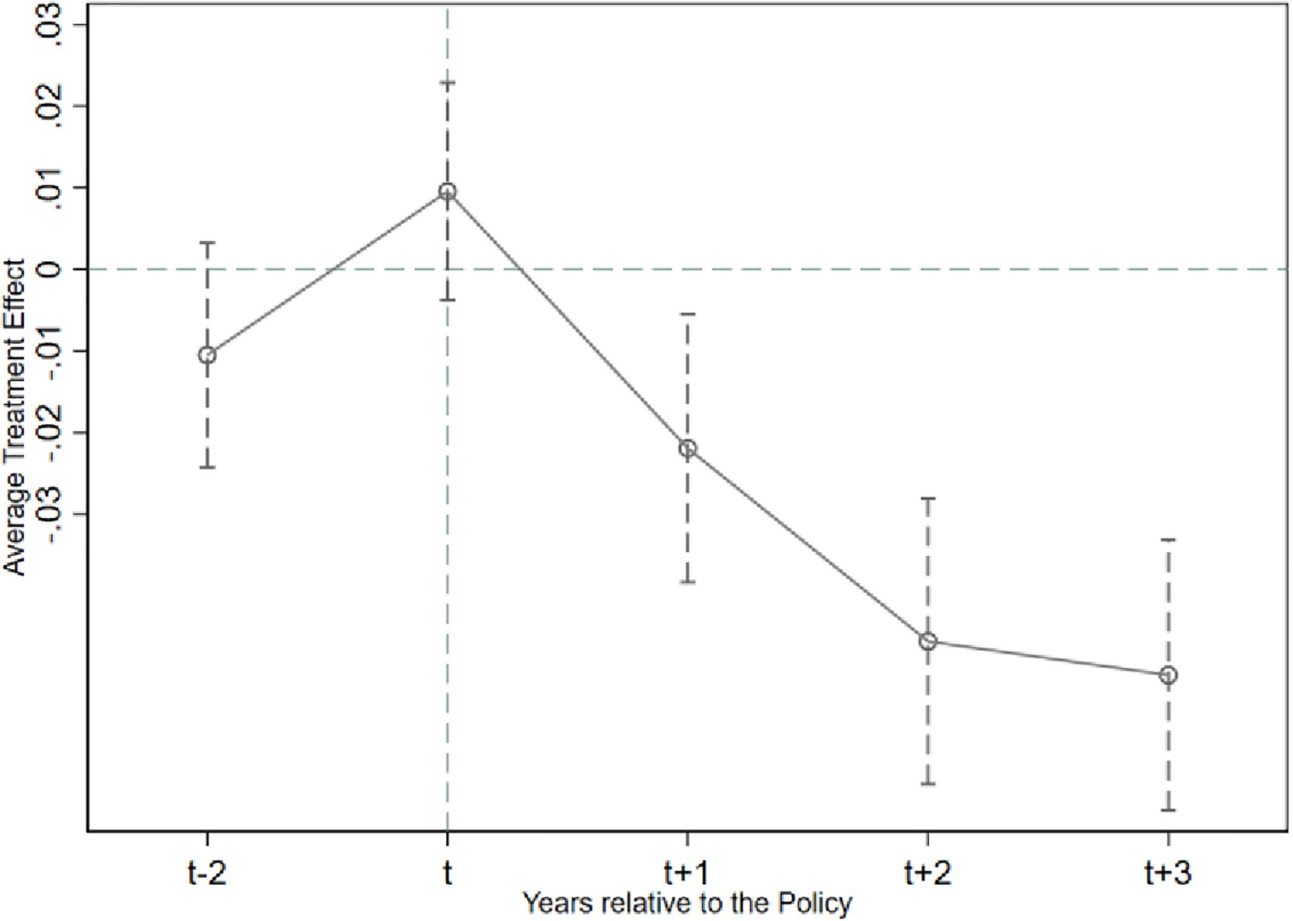
Plot of parallel trend test for *Cash*_*i*,*t*_.

**Fig 2:**
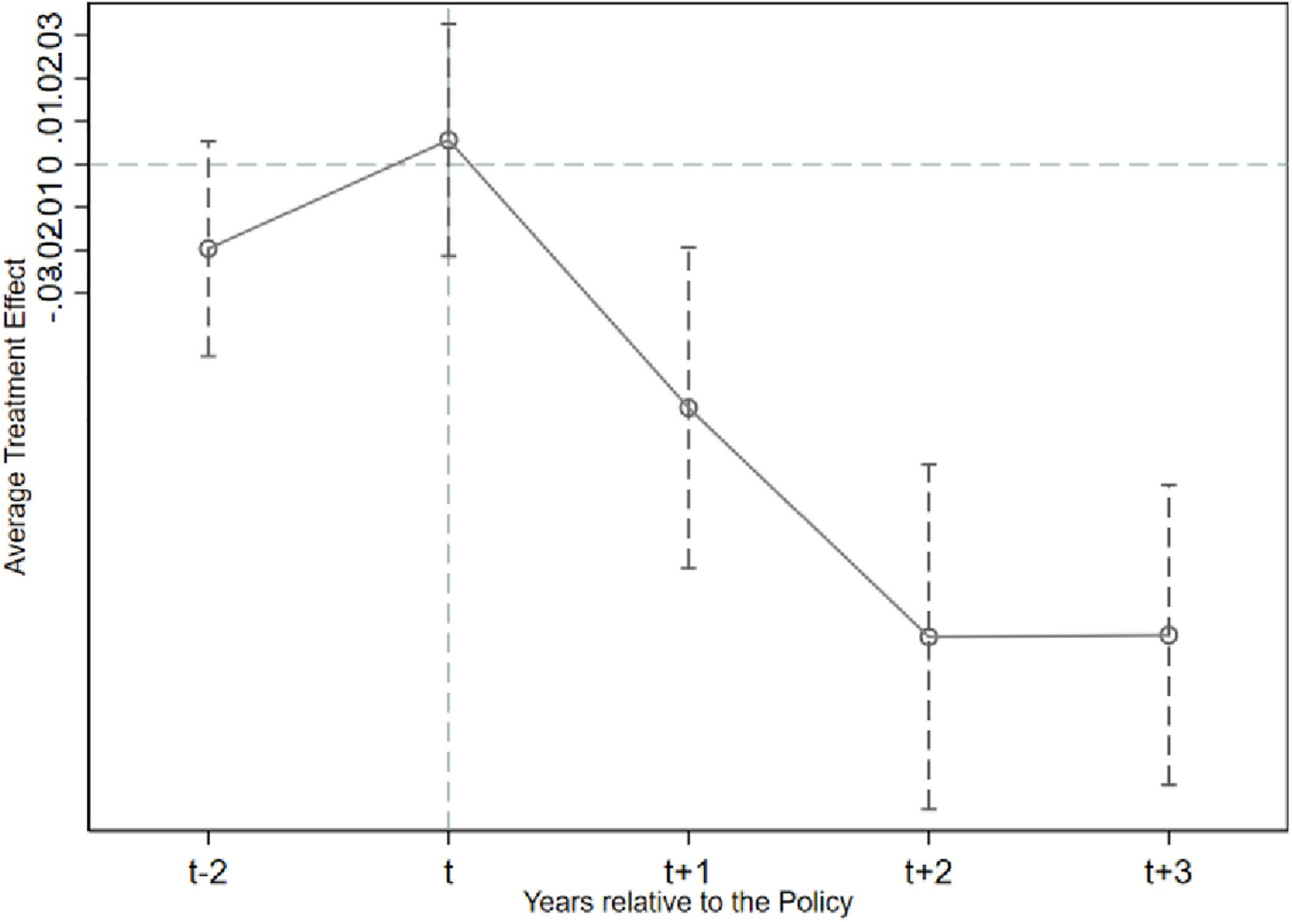
Plot of parallel trend test for *Cash_a*_*i*,*t*_.

The results of the parallel trend test show that the conclusions of this research satisfy the hypotheses related to the parallel trend test. We also plotted parallel trends in corporate cash holdings. Details are shown in Figs 1 and 2 below.

#### 4.4.2. The following robustness tests and discussions were carried out to further guarantee the accuracy of the paper’s conclusions

Substitution of measurements for explanatory factors. This research conducts robustness testing by substituting the explanatory variable to reduce the impact of variable measure errors on the empirical findings (*TP*_*i*,*t*_). As a result of the different levels of classification among China’s state-owned enterprises, they are distributed at the provincial, municipal, and lower levels, with provincial and central-level enterprises typically being larger in scale and occupying a dominant position in the economy. The distinction between the two is derived from the information disclosed on China’s stock market, which reveals the level of state-owned enterprises. Using the level classification of state-owned enterprises in the CSMAR database, we ultimately classified state-owned enterprises accordingly. Specifically, we redefine the grouping basis of the experimental group by designating only central and above-provincial SOEs as the new pay control group (*SOE_m*_*i*,*t*_), while lower-tier SOEs are redefined as the control group, and we regress the interaction term *TP_m*_*i*,*t*_ by including it in the model (1). The findings of this robustness test are presented in Table 6. The findings in Table 6 are in line with the benchmark regression, demonstrating the validity of the findings of this study and the substantial decrease in corporate cash holdings caused by the "Salary Limitation Order".

**Table 6:** Change in the way the compensation control group is defined.

	(1)	(2)
	*Cash*_*i*,*t*_	*Cash_a*_*i*,*t*_
*TP_m*_*i*,*t*_	-0.0161**	-0.0391***
	(-2.5328)	(-2.8294)
*SOE_m*_*i*,*t*_	-0.0036	0.0151
	(-0.5009)	(1.0181)
*Size*_*i*,*t*_	-0.0203***	-0.0502***
	(-7.3794)	(-8.8109)
*Lev*_*i*,*t*_	0.1127***	0.1569***
	(7.7523)	(5.4395)
*ROA*_*i*,*t*_	0.5876***	1.2964***
	(10.5273)	(9.7610)
*Growth*_*i*,*t*_	-0.9286**	-2.5206***
	(-2.5700)	(-3.2902)
*Audit*_*i*,*t*_	0.0242*	0.0314
	(1.8889)	(1.4819)
*Board*_*i*,*t*_	0.0088	0.0018
	(0.6833)	(0.0679)
*Wage*3_*i*,*t*_	0.0199***	0.0396***
	(4.6887)	(4.4590)
*TOP*1_*i*,*t*_	0.0006***	0.0014***
	(3.4486)	(3.4796)
Cons	0.1768**	0.5384***
	(2.4947)	(3.8468)
N	5926	5926
Year	Yes	Yes
Industry	Yes	Yes
adj. R2	0.1936	0.1536

Note: The standard errors are corrected for clustering at the firm level.

***, **, and * indicate significance at the 1%, 5%, and 10% levels, respectively; t-values are given in parentheses.

#### 4.4.3. Matching using propensity scores

To improve the robustness of testing and reduce variability between the experimental and control groups, this study employs the PSM-DID approach and uses radius matching as the specific matching technique. Matching variables were determined using Psestimate results, and covariates such as firm size *Size*_*i*,*t*_), financial leverage (*Lev*_*i*,*t*_), firm profitability (*ROA*_*i*,*t*_, board size (*Board*_*i*,*t*_), and equity concentration (*TOP*1_*i*,*t*_) were selected. The bias of the matched samples was greatly reduced, indicating no significant difference between the experimental and control groups, which satisfies the conditions of the propensity score matching approach (unreported results).

The given regression results, as presented in Table 7, indicate that the coefficients of the interaction term *TP*_*i*,*t*_ are negative, at -0.0258 and -0.0661, respectively, after conducting PSM matching. These coefficients were found to be statistically significant at the 1% level. Importantly, subsequent robustness tests using the PSM-DID method reveal that the coefficient of the interaction term *TP*_*i*,*t*_ remains consistent with the benchmark regression. Consequently, the study’s conclusions remain valid. These findings suggest that the results of the study are robust and lend credence to the reliability of the study’s conclusions.

**Table 7:** PSM-DID matching results.

	(1)	(2)
	*Cash*_*i*,*t*_	*Cash_a*_*i*,*t*_
*TP*_*i*,*t*_	-0.0258***	-0.0661***
	(-4.0338)	(-4.9142)
*SOE*_*i*,*t*_	-0.0018	0.0204
	(-0.2596)	(1.5688)
*Size*_*i*,*t*_	-0.0192***	-0.0480***
	(-6.9545)	(-8.5357)
*Lev*_*i*,*t*_	0.1146***	0.1590***
	(7.9091)	(5.5613)
*ROA*_*i*,*t*_	0.5845***	1.2960***
	(10.3468)	(9.5013)
*Growth*_*i*,*t*_	-0.9644***	-2.5565***
	(-2.6489)	(-3.3278)
*Audit*_*i*,*t*_	0.0235*	0.0300
	(1.8175)	(1.4380)
*Board*_*i*,*t*_	0.0121	0.0070
	(0.9288)	(0.2546)
*Wage*3_*i*,*t*_	0.0196***	0.0389***
	(4.6690)	(4.4045)
*TOP*1_*i*,*t*_	0.0007***	0.0014***
	(3.4690)	(3.5491)
Cons	0.1531**	0.4973***
	(2.1301)	(3.4922)
N	5914	5914
Year	Yes	Yes
Industry	Yes	Yes
adj. R2	0.1980	0.1576

Note: The standard errors are corrected for clustering at the firm level.

***, **, and * indicate significance at the 1%, 5%, and 10% levels, respectively; t-values are given in parentheses.

#### 4.4.4. Placebo test

**Fig 3:**
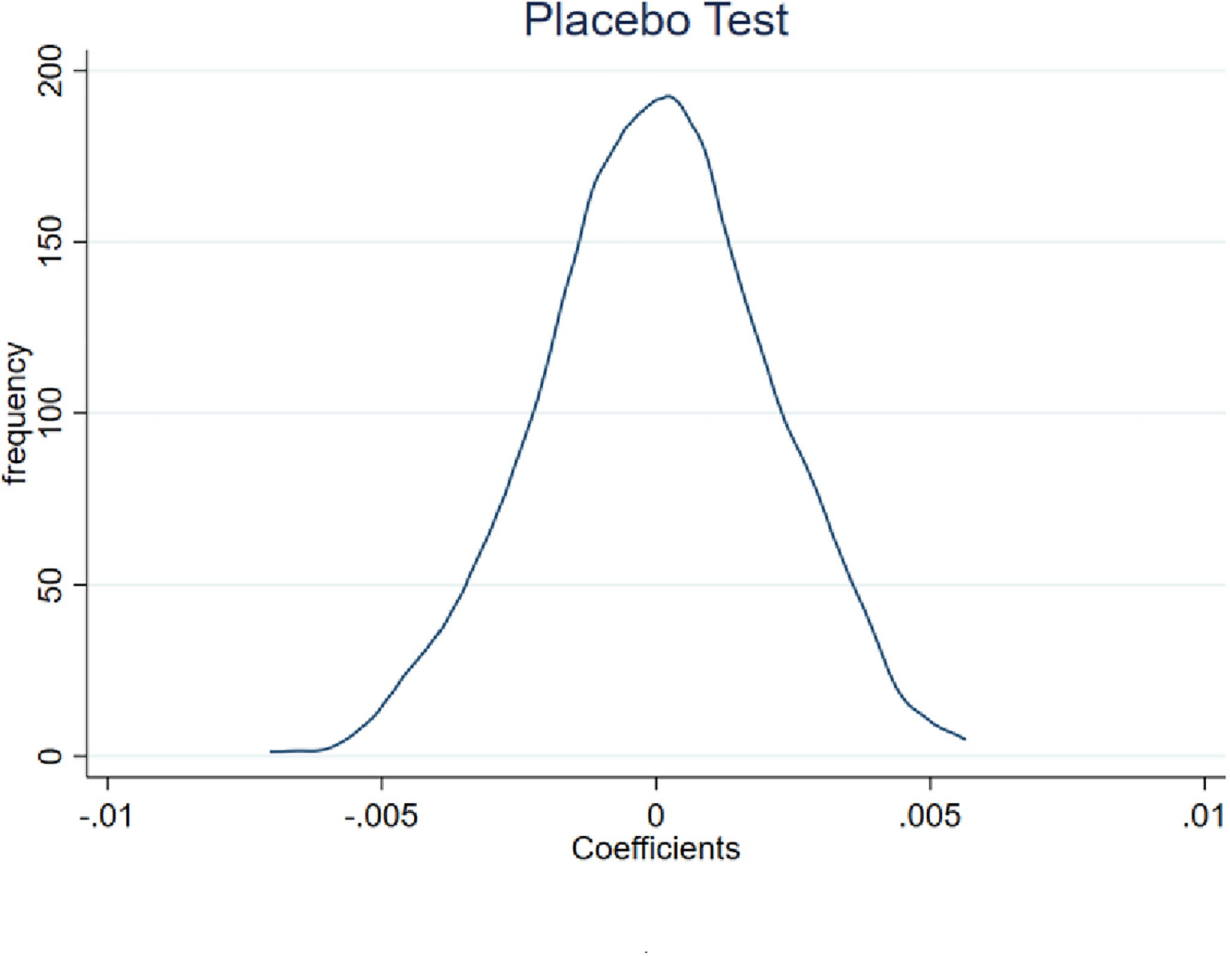
Distribution of coefficients on *Cash*_*i*,*t*_ after 1000 randomizations of the "Salary Limitation Order".

**Fig 4:**
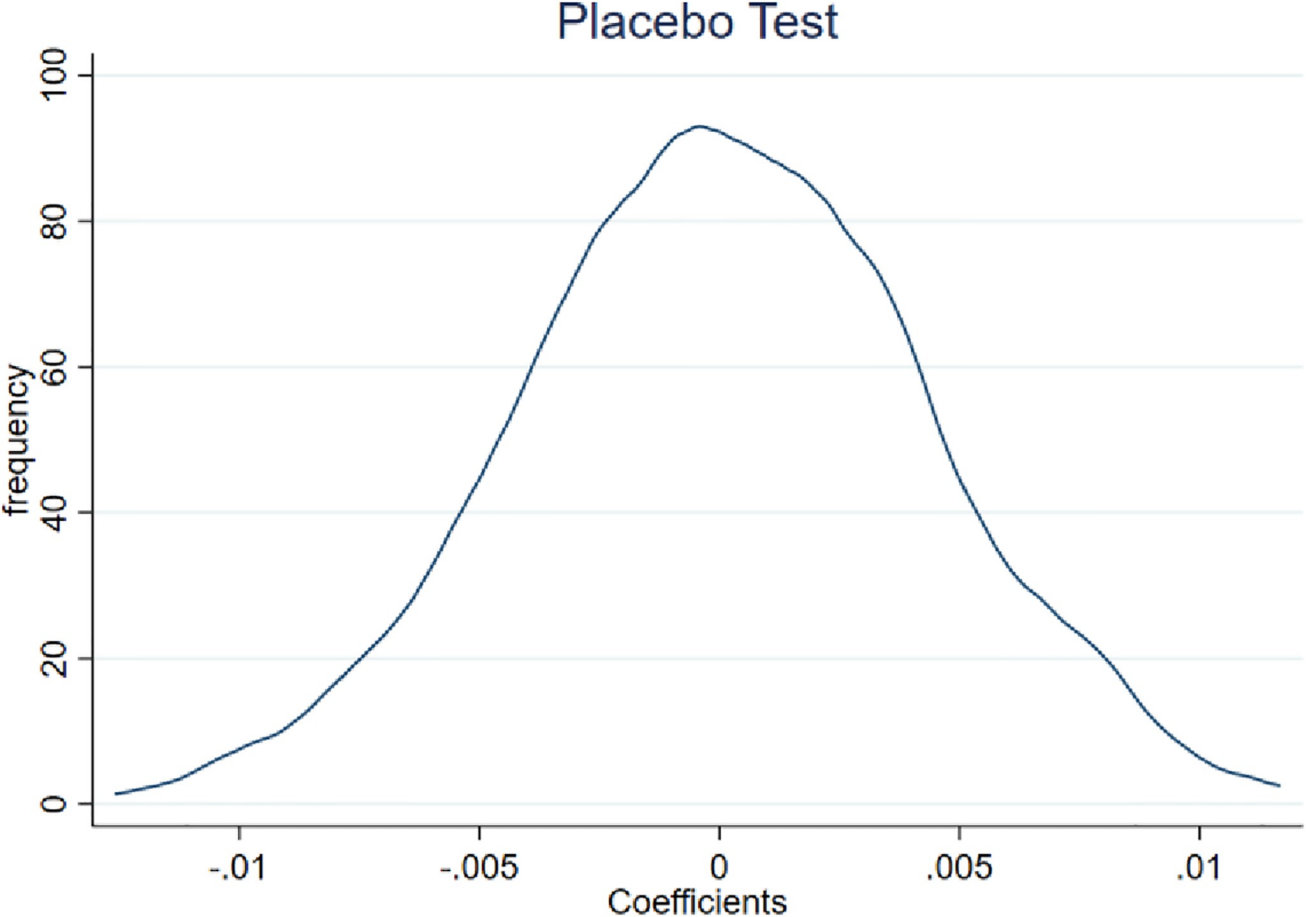
Distribution of coefficients on *Cash_a*_*i*,*t*_ after 1000 randomizations of the "Salary Limitation Order".

By randomly dividing the treatment and control groups into two groups, a placebo test was employed to rule out the possibility that the results of this research are the result of other unobservable factors, drawing on Li et al [48]. By creating a random dummy variable for "Salary Limitation Order" in the entire sample, regressing it using the regression model in model (1), and repeating the randomization procedure 1000 times, we specifically randomize the effect of "payroll restriction" on SOEs. The constraint effect of "Salary Limitation Order" on SOEs is no longer substantial after the randomization procedure. The distribution of the coefficients for *Cash*_*i*,*t*_ and *Cash_a*_*i*,*t*_ is shown in Figs 3 and 4, respectively. The fact that the payroll control coefficients are concentrated at zero suggests that the conclusions of this research are not the result of unobservable factors.

## 5. Further Research

### 5.1. Mechanism test analysis

Building upon the preceding description, the present study aims to examine whether the agent cost assumes the role of an intermediary. To this end, the management expense ratio (*Fee*_*i*,*t*_) is utilized as one of the indicators to gauge the magnitude of agent costs. Drawing inspiration from the approach outlined by Baron and Kenny, the current investigation expands upon model (1) and incorporates models (2) and (3) to evaluate potential mediation effects [49].


$$Fee_{i,t}=\beta_{0}+\beta_{1}TP_{i,t}+\beta_{2}SOE_{i,t}+\beta_{i}Controls_{i,t}+\sum Year+\sum Industry+\varepsilon_{i,t}$$ (2)

$$Cash_{i,t}/Cash\_a_{i,t}=\beta_{0}+\beta_{1}TP_{i,t}+\beta_{2}SOE_{i,t}+\beta_{3}Fee_{i,t}+\beta_{i}Controls_{i,t}+\sum Year+\sum Industry+\varepsilon_{i,t}$$ (3)

The regression results are presented in Table 8, where the negative and significant coefficient of the interaction term *TP*_*i*,*t*_ in columns (1) and (2) demonstrates a negative correlation between the "Salary Limitation Order" and SOE cash holdings. In column (3), the negative coefficient of the interaction term *TP*_*i*,*t*_ at the 5% level reveals some inhibitory effects of the payroll control on the firm’s agency cost *Fee*_*i*,*t*_. Columns (4) and (5) examine the coefficients of the interaction term TP_i,t_ and the agency cost Fee_i,t_, indicating one positive and one negative coefficient for each. Both coefficients of the agency cost *Fee*_*i*,*t*_, 0.1543 and 0.5550, are significant at the 1% level, as are the coefficients of the interaction term *TP*_*i*,*t*_, which are -0.0248 and -0.0621. These findings suggest that agency cost *Fee*_*i*,*t*_ partly mediates the decline in cash holdings, and that it is a significant factor in CEO decisions to hold cash and in compensation constraints affecting company cash holdings. The hypothesis H2 holds.

**Table 8:** Mechanism test regression results.

	(1)	(2)	(3)	(4)	(5)
	*Cash*_*i*,*t*_	*Cash_a*_*i*,*t*_	*Fee*_*i*,*t*_	*Cash*_*i*,*t*_	*Cash_a*_*i*,*t*_
*TP*_*i*,*t*_	-0.0259***	-0.0661***	-0.0072**	-0.0248***	-0.0621***
	(-4.0523)	(-4.9160)	(-2.5396)	(-3.8827)	(-4.6357)
*Fee*_*i*,*t*_				0.1543***	0.5550***
				(2.7344)	(3.9720)
*SOE*_*i*,*t*_	-0.0019	0.0199	0.0075**	-0.0030	0.0157
	(-0.2741)	(1.5252)	(1.9963)	(-0.4432)	(1.1909)
*Size*_*i*,*t*_	-0.0191***	-0.0477***	-0.0199***	-0.0161***	-0.0367***
	(-6.9411)	(-8.4951)	(-15.2755)	(-5.4417)	(-6.0335)
*Lev*_*i*,*t*_	0.1137***	0.1576***	-0.0425***	0.1203***	0.1812***
	(7.8375)	(5.5036)	(-5.6266)	(8.2203)	(6.2507)
*ROA*_*i*,*t*_	0.5752***	1.2765***	-0.0405	0.5814***	1.2990***
	(10.1417)	(9.3622)	(-1.5296)	(10.2354)	(9.5403)
*Growth*_*i*,*t*_	-0.9837***	-2.6014***	-1.5226***	-0.7487**	-1.7563**
	(-2.7012)	(-3.3822)	(-7.7961)	(-1.9814)	(-2.1841)
*Audit*_*i*,*t*_	0.0238*	0.0307	-0.0332***	0.0290**	0.0491**
	(1.8493)	(1.4778)	(-3.0480)	(2.1470)	(2.1596)
*Board*_*i*,*t*_	0.0125	0.0088	-0.0109*	0.0142	0.0148
	(0.9620)	(0.3223)	(-1.7963)	(1.0870)	(0.5404)
*Wage*3_*i*,*t*_	0.0201***	0.0397***	0.0088***	0.0187***	0.0348***
	(4.7467)	(4.4780)	(4.6177)	(4.3904)	(3.9511)
*TOP*1_*i*,*t*_	0.0007***	0.0014***	-0.0003***	0.0007***	0.0016***
	(3.4888)	(3.5745)	(-3.4150)	(3.7154)	(3.9738)
Cons	0.1464**	0.4777***	0.4966***	0.0698	0.2021
	(2.0262)	(3.3449)	(14.0063)	(0.9071)	(1.2669)
N	5926	5926	5926	5926	5926
Year	Yes	Yes	Yes	Yes	Yes
Industry	Yes	Yes	Yes	Yes	Yes
adj. R2	0.1975	0.1568	0.2340	0.2007	0.1656

Note: The standard errors are corrected for clustering at the firm level.

***, **, and * indicate significance at the 1%, 5%, and 10% levels, respectively; t-values are given in parentheses.

### 5.2. Heterogeneity test

#### 5.2.1. compensation controls, internal controls, and corporate cash holdings

The present text examines the effects of compensation controls, internal controls, and corporate cash holdings. Notably, the efficacy of internal controls can influence the effectiveness of corporate governance mechanisms, which, in turn, can affect the financial decision-making of the firm. Specifically, our findings suggest that the impact of “Salary Limitation Order” on cash holdings is more pronounced in companies with lower internal control quality as compared to those with higher internal control quality. This is because companies with inferior internal control quality are more susceptible to agency problems, and managers, driven by self-interest, may have a stronger incentive to hold greater reserves of funds with weaker internal control quality [50]. The implementation of salary control can mitigate agency costs and is more effective in reducing excessive funds held by companies. Thus, the impact of “Salary Limitation Order” on cash holdings varies between companies with varying levels of internal control quality. We anticipate that companies with lower internal control quality will experience a more substantial reduction in cash holdings.

This article discusses the research approach adopted by Kuang Y.F. et al. and presents the results obtained through the utilization of the internal control index data from the China DIB database [50]. The data evaluates the quality of internal control of Chinese enterprises based on five aspects of the internal environment and calculates the average value of the same year and industry based on the internal control index. The samples are then divided into two groups based on their internal control index values, where samples with IC = 1 denote the high-quality internal control group, while those with IC = 0 denote the low-quality internal control group. The regression results presented in Table 9 indicate that the impact of compensation control on corporate cash holdings varies among samples with different internal control levels. Specifically, in columns (1) and (3), which correspond to the high-quality internal control group, the coefficient of the interaction term *TP*_*i*,*t*_ is -0.017 and -0.036, respectively, and both are significantly negative at the 10% level. In columns (2) and (4), which correspond to the low-quality internal control group, the coefficient of the interaction term *TP*_*i*,*t*_ is -0.037 and -0.099, respectively, and both are significantly negative at the 1% level. In addition to the aforementioned analysis, this study also conducted inter-group difference testing to further assess the comparability of the coefficients across different groups. Notably, the results of the inter-group difference testing demonstrate that the coefficients under scrutiny are indeed comparable across various groups, as evidenced by their successful passage of the aforementioned testing. Taken together, these findings suggest that the decrease in cash holdings is more significant when the internal control quality of an enterprise is low. Furthermore, the results indicate that the effect of compensation control on corporate cash holdings is contingent upon the level of internal control quality.

**Table 9:** Compensation controls, internal controls and corporate cash holdings.

	(1)	(2)	(3)	(4)
	IC = 1	IC = 0	IC = 1	IC = 0
	*Cash*_*i*,*t*_	*Cash*_*i*,*t*_	*Cash_a*_*i*,*t*_	*Cash_a*_*i*,*t*_
*TP*_*i*,*t*_	-0.017*	-0.037***	-0.036*	-0.099***
	(-1.805)	(-3.956)	(-1.818)	(-4.955)
*SOE*_*i*,*t*_	0.005	-0.007	0.031	0.013
	(0.532)	(-0.744)	(1.618)	(0.785)
*Size*_*i*,*t*_	-0.016***	-0.022***	-0.042***	-0.057***
	(-4.454)	(-6.067)	(-5.456)	(-7.536)
*Lev*_*i*,*t*_	0.151***	0.077***	0.230***	0.077**
	(8.138)	(4.046)	(6.553)	(2.019)
*ROA*_*i*,*t*_	0.700***	0.518***	1.570***	1.115***
	(7.134)	(8.736)	(6.241)	(8.692)
*Growth*_*i*,*t*_	-1.057**	-1.156**	-2.832***	-2.773**
	(-2.332)	(-1.977)	(-2.980)	(-2.176)
*Audit*_*i*,*t*_	0.035	0.023	0.095*	0.027
	(1.201)	(1.603)	(1.694)	(1.241)
*Board*_*i*,*t*_	0.001	0.026	-0.009	0.035
	(0.035)	(1.644)	(-0.246)	(1.092)
*Wage*3_*i*,*t*_	0.012**	0.026***	0.027**	0.048***
	(2.283)	(4.931)	(2.396)	(4.220)
*TOP*1_*i*,*t*_	0.001**	0.001***	0.001**	0.001***
	(2.207)	(3.213)	(2.525)	(2.941)
Cons	0.167*	0.129	0.394**	0.589***
	(1.758)	(1.366)	(2.000)	(3.077)
P-value	0.002***		0.000***	
N	2986	2938	2986	2938
Year	Yes	Yes	Yes	Yes
Industry	Yes	Yes	Yes	Yes
adj. R2	0.214	0.194	0.168	0.160

Note: The standard errors are corrected for clustering at the firm level.

***, **, and * indicate significance at the 1%, 5%, and 10% levels, respectively; t-values are given in parentheses.

#### 5.2.2. Compensation controls, management oversight, and corporate cash holdings

The management of a company is a composite of distinct rational individuals. The unilateral appropriation of a company’s rights by a manager is detrimental to the legitimate rights of other managers, and generates conflicts of interest within the management, as Ni X notes [26]. This article contends that the “Salary Limitation Order” has a greater impact on cash holdings in companies with stronger management supervision capabilities as compared to those with weaker ones. This is because weaker management supervision leads to less conspicuous internal conflicts of interest, and individual managers’ self-serving behavior will not be impeded or curtailed by other managers. Thus, even if the “Salary Limitation Order” is enforced, management in these companies will not be wholly compliant with regulations or make efforts to diminish their cash holdings. Conversely, companies with stronger management supervision capabilities experience a substantial reduction in agency costs and conflicts within the organization, when compared to those with weaker management supervision or poor organizational structures. Therefore, they are inclined to hold less cash. Therefore, when the “Salary Limitation Order” is implemented, it is expected that the internal governance level of executives in companies with stronger management supervision capabilities will improve, and they will be more prone to reduce their cash holdings [51]. Consequently, in companies with differing management supervision capabilities, this article anticipates that those with stronger supervision will considerably curtail their cash holdings.

This article utilizes the methodology developed by Ni X to gauge the management supervision ability through the management shareholding ratio [26]. The reasoning behind this approach is that a higher management shareholding ratio aligns the managers’ interests with those of the company, leading to a greater concern for actions that could potentially harm the company’s value. The sample is divided into two groups based on the annual and industry averages of the management shareholding ratio. Samples with a "Supervise" value of 1 correspond to the group with stronger management supervision ability, while samples with a "Supervise" value of 0 correspond to the group with weaker management supervision ability. The regression results are presented in Table 10, where columns (1) and (3) represent the regression results for the group with stronger management supervision ability, and columns (2) and (4) correspond to the weaker group. In column (1), the coefficient of the interaction term *TP*_*i*,*t*_ is -0.021, which is significant at the 5% level and negative. In contrast, in column (2), the coefficient of the interaction term *TP*_*i*,*t*_ is -0.005, which is not significant. In column (3), the coefficient of the interaction term *TP*_*i*,*t*_ is -0.055, which is significant at the 1% level and negative. However, in column (4), the coefficient of the interaction term *TP*_*i*,*t*_ is not significant with a value of -0.019. In addition to the aforementioned analysis, this study also conducted inter-group difference testing to further assess the comparability of the coefficients across different groups. Notably, the results of the inter-group difference testing demonstrate that the coefficients under scrutiny are indeed comparable across various groups, as evidenced by their successful passage of the aforementioned testing. These results suggest that stronger management supervision ability leads to a more significant reduction in cash holdings. Additionally, the findings suggest that the impact of salary control on cash holdings differs among companies with varying levels of management supervision ability.

**Table 10:** Compensation controls, management oversight, and corporate cash holdings.

	(1)	(2)	(3)	(4)
	Supervise = 1	Supervise = 0	Supervise = 1	Supervise = 0
	*Cash*_*i*,*t*_	*Cash*_*i*,*t*_	*Cash_a*_*i*,*t*_	*Cash_a*_*i*,*t*_
*TP*_*i*,*t*_	-0.021**	-0.005	-0.055***	-0.019
	(-2.357)	(-0.462)	(-2.805)	(-0.925)
*SOE*_*i*,*t*_	0.008	-0.016	0.047**	-0.016
	(0.908)	(-1.590)	(2.470)	(-0.856)
*Size*_*i*,*t*_	-0.028***	-0.009**	-0.070***	-0.024***
	(-7.204)	(-2.494)	(-8.488)	(-3.440)
*Lev*_*i*,*t*_	0.083***	0.133***	0.099**	0.193***
	(4.108)	(7.117)	(2.351)	(5.678)
*ROA*_*i*,*t*_	0.622***	0.510***	1.406***	1.106***
	(7.921)	(7.705)	(7.217)	(7.270)
*Growth*_*i*,*t*_	-0.965*	-1.072**	-3.121**	-2.290**
	(-1.725)	(-2.365)	(-2.552)	(-2.443)
*Audit*_*i*,*t*_	-0.005	0.032**	-0.013	0.038*
	(-0.268)	(2.225)	(-0.353)	(1.890)
*Board*_*i*,*t*_	0.001	0.033*	-0.023	0.062*
	(0.052)	(1.842)	(-0.630)	(1.830)
*Wage*3_*i*,*t*_	0.021***	0.017***	0.041***	0.034***
	(3.731)	(3.072)	(3.385)	(2.875)
*TOP*1_*i*,*t*_	0.001***	0.000	0.002***	0.001
	(4.170)	(1.385)	(3.939)	(1.328)
Cons	0.401***	-0.103	1.069***	-0.092
	(4.129)	(-1.070)	(5.185)	(-0.508)
P-value	0.015**		0.004***	
N	3164	2762	3164	2762
Year	Yes	Yes	Yes	Yes
Industry	Yes	Yes	Yes	Yes
adj. R2	0.230	0.167	0.180	0.137

Note: The standard errors are corrected for clustering at the firm level.

***, **, and * indicate significance at the 1%, 5%, and 10% levels, respectively; t-values are given in parentheses.

#### 5.2.3. Compensation regulation, degree of information asymmetry, and corporate cash holdings

Asymmetric information represents one of the significant factors that influence agency problems, and the retention of excessive cash by management for on-the-job consumption constitutes a classic example of moral hazard. In companies with lower levels of information asymmetry, the magnitude of agency conflicts may be smaller, and the efficacy of corporate governance mechanisms may be greater. The present article posits that, compared to companies with higher levels of information asymmetry, the influence of the "Salary Limitation Order" on cash holdings is more pronounced in companies with lower levels of information asymmetry. This is because companies with lower levels of information asymmetry tend to have less severe agency problems, which makes the governance mechanisms of the company more effective. When it comes to the governance of salary control, effective disclosure of management compensation levels can help reduce information asymmetry. As users of financial statement information, shareholders can also gain a comprehensive understanding of the company’s compensation structure, which further reduces agency costs. Consequently, the reduction in cash holdings caused by salary control will be more significant [52]. Thus, the impact of the "Salary Limitation Order" on cash holdings varies depending on the level of information asymmetry present in different companies. The present article anticipates that companies with lower levels of information asymmetry will experience a more significant reduction in cash holdings. This paper employs the modified Jones model to compute the extent of information asymmetry pertaining to manipulative accruals profitability measures. Specifically, annual and industry-specific regressions are conducted using the model specified in

$$\frac{TA_{i,t}}{Asset_{i,t-1}}=\alpha_{1}\frac{1}{A{\text{sset}}_{i,t-1}}+\alpha_{2}\frac{\Delta REV_{i,t}}{Asset_{i,t-1}}+\alpha_{3}\frac{PPE_{i,t}}{Asset_{i,t-1}}+\varepsilon_{i,t}$$

The estimated regression coefficients are then incorporated into the formula denoted by

$$\begin{array}{l} DA_{i,t}=\frac{TA_{i,t}}{Aset_{i,t-1}}-({\hat{\alpha }}_{1}\frac{1}{Asset_{i,t-1}}+{\hat{\alpha }}_{2}\frac{\Delta REV_{i,t}-\Delta REC_{i,t}}{Asset_{i,t-1}}+{\hat{\alpha }}_{3}\frac{PPE_{i,t}}{Aset_{i,t-1}}) \end{array}$$

to derive an estimate of manipulative accruals profitability. Subsequently, the research sample is divided into two groups based on the mean manipulative accruals profitability for each year and industry. Samples with Dis_acc = 1 are indicative of a higher degree of information asymmetry, while Dis_acc = 0 represents a lower degree of information asymmetry. Regression results are presented in Table 11, with columns (1) (3) and columns (2) (4) corresponding to regression outcomes for the higher and lower information asymmetry groups, respectively. In column (1), the coefficient of the interaction term *TP*_*i*,*t*_ is -0.016, which is statistically significant at the 10% level. In column (2), the coefficient of the interaction term *TP*_*i*,*t*_ is -0.033, which is statistically significant at the 1% level. In column (3), the coefficient of the interaction term *TP*_*i*,*t*_ is -0.044, which is statistically significant at the 5% level. In column (4), the coefficient of the interaction term *TP*_*i*,*t*_ is -0.082, which is statistically significant at the 1% level. In addition to the aforementioned analysis, this study also conducted inter-group difference testing to further assess the comparability of the coefficients across different groups. Notably, the results of the inter-group difference testing demonstrate that the coefficients under scrutiny are indeed comparable across various groups, as evidenced by their successful passage of the aforementioned testing. This indicates that when a company has a higher level of information asymmetry, the impact of compensation control on cash holdings is more pronounced. Additionally, it suggests that the effect of compensation control on cash holdings varies across samples with differing levels of information asymmetry.

**Table 11:** Compensation regulation, degree of information asymmetry, and corporate cash holdings.

	(1)	(2)	(3)	(4)
	Dis_acc = 1	Dis_acc = 0	Dis_acc = 1	Dis_acc = 0
	*Cash*_*i*,*t*_	*Cash*_*i*,*t*_	*Cash_a*_*i*,*t*_	*Cash_a*_*i*,*t*_
*TP*_*i*,*t*_	-0.016*	-0.033***	-0.044**	-0.082***
	(-1.688)	(-3.597)	(-2.235)	(-4.167)
*SOE*_*i*,*t*_	-0.007	0.002	0.014	0.023
	(-0.789)	(0.269)	(0.735)	(1.476)
*Size*_*i*,*t*_	-0.015***	-0.022***	-0.038***	-0.054***
	(-4.660)	(-6.721)	(-5.843)	(-7.810)
*Lev*_*i*,*t*_	0.130***	0.098***	0.185***	0.128***
	(7.286)	(5.375)	(5.271)	(3.411)
*ROA*_*i*,*t*_	0.465***	0.753***	0.969***	1.818***
	(7.560)	(8.785)	(7.503)	(8.059)
*Growth*_*i*,*t*_	-0.791*	-1.054*	-1.550*	-3.402**
	(-1.727)	(-1.690)	(-1.756)	(-2.419)
*Audit*_*i*,*t*_	0.033***	0.011	0.054***	0.005
	(2.840)	(0.444)	(2.757)	(0.127)
*Board*_*i*,*t*_	0.016	0.006	0.027	-0.018
	(1.026)	(0.381)	(0.842)	(-0.486)
*Wage*3_*i*,*t*_	0.020***	0.021***	0.037***	0.043***
	(4.007)	(4.068)	(3.710)	(3.704)
*TOP*1_*i*,*t*_	0.001**	0.001***	0.001*	0.002***
	(2.401)	(3.170)	(1.918)	(3.517)
Cons	0.035	0.229***	0.239	0.670***
	(0.406)	(2.668)	(1.443)	(3.698)
P-value	0.004***		0.001***	
N	2932	2880	2932	2880
Year	Yes	Yes	Yes	Yes
Industry	Yes	Yes	Yes	Yes
adj. R2	0.164	0.233	0.124	0.197

Note: The standard errors are corrected for clustering at the firm level.

***, **, and * indicate significance at the 1%, 5%, and 10% levels, respectively; t-values are given in parentheses.

## 6. Conclusion

The interest and sensitivity towards pay regulation among various stakeholders have significantly increased since the introduction of China’s "Guidance on Further Regulating the Management of Remuneration for Heads of Central Enterprises." However, limited research has been conducted on agency costs, cash holdings, and compensation regulation. This study uses Chinese listed companies from 2007 to 2012 as the research sample, with state-owned enterprises as the experimental group and non-state-owned enterprises as the control group. The study aims to investigate the impact of compensation regulation implementation on firms’ cash holdings by conducting a quasi-natural experiment in 2009, coinciding with the implementation of China’s "Guidance on Further Regulating the Management of Remuneration for Heads of Central Enterprises". Through this study, we aim to contribute to the research on the financial effects of pay regulation.

Payroll regulation considerably affects SOEs’ cash holdings, according to the findings of this study. This conclusion was maintained after utilizing the parallel trend test, replacing the experimental group, and using the PSM-DID approach for robustness testing. Agency expenses have a limited mediating role in the regulation of compensation and corporate cash holdings. According to heterogeneity analysis, the sample with the best internal control, the most effective management monitoring, and the least information asymmetry had the greatest impact of compensation legislation on corporate cash holdings. These results imply that, when seen from many angles, the effect of "Salary Limitation Order" on corporate cash holdings is still relevant.

The study’s findings offer the following crystal-clear advice for businesses and shareholders: The influence of conflicts of interest brought on by agency issues should be minimized, and companies should pay attention to the power of internal controllers. To prevent management self-interest from the start, businesses should take the initiative to upgrade their internal governance processes. To eliminate knowledge asymmetry within the organization and to emphasize the incentive role of remuneration, businesses should deliberately construct incentive compensation contracts. Managers should, in turn, concentrate on the business operations of the company and improve the financial impact of the pay contract on the company.

This paper does have some restrictions. First, there could be bias in the measurement and selection of the variables used in this publication, which could have an impact on the study’s findings. Second, the sample selection may have been constrained. While many non-listed Chinese companies are not included in the study sample and may have certain sample constraints, this paper uses Chinese-listed companies as its research sample. If pertinent data support this in future investigations, we will improve this.

## References

[1] Lazear E. P., & Rosen S. (1981). Rank-order tournaments as optimum labor contracts. Journal of Political Economy, 89(5), 841–864.

[2] Conyon M. J., Peck S. I., & Sadler G. V. (2001). Corporate tournaments and executive compensation: Evidence from the UK. Strategic Management Journal, 22(8), 805–815.

[3] Rosen S. (1986). The theory of equalizing differences. Handbook of labor economics, 1, 641–692.

[4] Cowherd D. M., & Levine D. I. (1992). Product quality and pay equity between lower-level employees and top management: An investigation of distributive justice theory. Administrative Science Quarterly, 302–320.

[5] Williams M. L. (1995). Antecedents of employee benefit level satisfaction: A test of a model. Journal of Management, 21(6), 1097–1128.

[6] Carpenter M. A., & Sanders W. G. (2004). The effects of top management team pay and firm internationalization on MNC performance. Journal of Management, 30(4), 509–528.

[7] Williams M. L., McDaniel M. A., & Nguyen N. T. (2006). A meta-analysis of the antecedents and consequences of pay level satisfaction. Journal of Applied Psychology, 91(2), 392. doi: 10.1037/0021-9010.91.2.392 16551191

[8] Lambert R. A., Larcker D. F., & Weigelt K. (1993). The structure of organizational incentives. Administrative Science Quarterly, 438–461.

[9] Main B. G., O’Reilly C. A. III, & Wade J. (1993). Top executive pay: Tournament or teamwork?. Journal of Labor Economics, 11(4), 606–628.

[10] Erickson M., & Wang S. W. (1999). Earnings management by acquiring firms in stock for stock mergers. Journal of Accounting and Economics, 27(2), 149–176.

[11] Mehran H. (1995). Executive compensation structure, ownership, and firm performance. Journal of Financial Economics, 38(2), 163–184.

[12] Adams J. S. (1963). Towards an understanding of inequity. The journal of abnormal and social psychology, 67(5), 422. 10.1037/h0040968 14081885

[13] Grund C., & Sliwka D. (2005). Envy and compassion in tournaments. Journal of Economics & Management Strategy, 14(1), 187–207.

[14] Hayward M. L., & Hambrick D. C. (1997). Explaining the premiums paid for large acquisitions: Evidence of CEO hubris. Administrative science quarterly, 103–127.

[15] Chhaochharia V., & Grinstein Y. (2007). Corporate governance and firm value: The impact of the 2002 governance rules. Journal of Finance, 62(4), 1789–1825.

[16] Jensen M. C., & Murphy K. J. (1990). Performance pay and top-management incentives. Journal of Political Economy, 98(2), 225–264.

[17] Thanassoulis J. (2012). The case for intervening in bankers’ pay. Journal of Finance, 67(3), 849–895.

[18] Frydman C., & Molloy R. (2012). Pay cuts for the boss: Executive compensation in the 1940s. The Journal of Economic History, 72(1), 225–251.

[19] Cebon P., & Hermalin B. E. (2015). When less is more: The benefits of limits on executive pay. Review of Financial Studies, 28(6), 1667–1700.

[20] Kessler A. (2009). Bank Pay Controls Aren’t the Answer. Wall Street Journal.

[21] Opler T., Pinkowitz L., Stulz R., & Williamson R. (1999). The determinants and implications of corporate cash holdings. Journal of Financial Economics, 52(1), 3–46.

[22] Bates T. W., Kahle K. M., & Stulz R. M. (2009). Why do US firms hold so much more cash than they used to? Journal of Finance, 64(5), 1985–2021.

[23] Duchin R. (2010). Cash holdings and corporate diversification. Journal of Finance, 65(3), 955–992.

[24] Myers S. C., & Rajan R. G. (1998). The paradox of liquidity. The Quarterly Journal of Economics, 113(3), 733–771.

[25] Richardson S. (2006). Over-investment of free cash flow. Review of Accounting Studies, 11(2), 159–189.

[26] Ni X. Does stakeholder orientation matter for earnings management: Evidence from non-shareholder constituency statutes[J]. Journal of Corporate Finance, 2020, 62: 101606.

[27] Shleifer A., & Vishny R. W. (1997). A survey of corporate governance. Journal of Finance, 52(2), 737–783.

[28] Jensen M. C. (1986). Agency costs of free cash flow, corporate finance, and takeovers. The American Economic Review, 76(2), 323–329.

[29] Cheng Y., Harford J., Hutton I., Shipe S. Ex Post Bargaining, Corporate Cash Holdings, and Executive Compensation (2022) Journal of Financial and Quantitative Analysis, 57 (3), pp. 957–987.

[30] Blanchard O. J., Lopez-de-Silanes F., & Shleifer A. (1994). What do firms do with cash windfalls?. Journal of Financial Economics, 36(3), 337–360.

[31] Jensen M. C., & Meckling W. H. (1976). Theory of the firm: Managerial behavior, agency costs and ownership structure. Journal of Financial Economics, 3(4): 305–360.

[32] Dittmar A., & Mahrt-Smith J. (2007). Corporate governance and the value of cash holdings. Journal of Financial Economics, 83(3), 599–634.

[33] Harford J., Mansi S. A., & Maxwell W. F. (2008). Corporate governance and firm cash holdings in the US. Journal of Financial Economics, 87(3), 535–555.

[34] Kalcheva I., & Lins K. V. (2007). International evidence on cash holdings and expected managerial agency problems. Review of Financial Studies, 20(4), 1087–1112.

[35] Lee C.-H., Chou P.-I. Corporate cash holdings and product market competition: The effects of stock-based executive compensation (2018) Asian Economic and Financial Review, 8 (9), pp. 1140–1157.

[36] Dimitropoulos P., Koronios K., Thrassou A., Vrontis D. Cash holdings, corporate performance and viability of Greek SMEs: Implications for stakeholder relationship management (2020) EuroMed Journal of Business, 15 (3), pp. 333–348.

[37] Faulkender M., & Wang R. (2006). Corporate financial policy and the value of cash. Journal of Finance, 61(4), 1957–1990.

[38] Frésard L., & Salva C. (2010). The value of excess cash and corporate governance: Evidence from US cross-listings. Journal of Financial Economics, 98(2), 359–384.

[39] Cao X., Lemmon M., Pan X., Qian M., and Tian G. 2019.“Political Promotion, CEO Incentives, and the Relationship Between Pay and Performance.” Management Science 65 (7): 2947–2965.

[40] Jiang F., and Kim K. A. 2020. “Corporate Governance inChina: A Survey.” Review of Finance 24 (4): 733–772.

[41] Hui Z. Y. and Fang H. Y. (2022) "Government controlling ownership and CEO compensation incentives: evidence from China." Applied Economics.

[42] Ozkan A., & Ozkan N. (2004). Corporate cash holdings: An empirical investigation of UK companies. Journal of Banking & Finance, 28(9), 2103–2134.

[43] Nyborg K. G., & Wang Z. (2021). The effect of stock liquidity on cash holdings: The repurchase motive. Journal of Financial Economics, 142(2), 905–927.

[44] Begenau J., & Palazzo B. (2021). Firm selection and corporate cash holdings. Journal of Financial Economics, 139(3), 697–718.

[45] Eskandari R., & Zamanian M. (2022). Cost of carry, financial constraints, and dynamics of corporate cash holdings. Journal of Corporate Finance, 102216.

[46] Chang Y., Pan X., Wang J., & Zhou Q. (2021). Depoliticization and corporate cash holdings: Evidence from the mandated resignation of directors in China. Journal of Corporate Finance, 69, 102004.

[47] Beuselinck C., Markarian G., & Verriest A. (2021). Employee protection shocks and corporate cash holdings. Journal of Corporate Finance, 69, 102027.

[48] Li P., Lu Y., & Wang J. (2016). Does flattening government improve economic performance? Evidence from China. Journal of Development Economics, 123, 18–37.

[49] Baron R. M., & Kenny D. A. (1986). The moderator–mediator variable distinction in social psychological research: Conceptual, strategic, and statistical considerations. Journal of Personality and Social Psychology, 51(6), 1173. doi: 10.1037//0022-3514.51.6.1173 3806354

[50] Kuang Y F, Lee G, Qin B. Whistleblowing allegations, audit fees, and internal control deficiencies[J]. Contemporary Accounting Research, 2021, 38(1): 32–62.

[51] Kontus E. (2021). Agency costs, capital structure and corporate performance: a survey of croatian, slovenian and czech listedcompanies. Ekonomski Vjesnik, 34(1), 73–85.

[52] Jiang F, Zhu B, Huang J. CEO’s financial experience and earnings management[J]. Journal of Multinational Financial Management, 2013, 23(3): 134–145.

